# Instability resistance training in collegiate basketball: a multitheoretical experimental study

**DOI:** 10.3389/fspor.2026.1827231

**Published:** 2026-05-28

**Authors:** Hengyu Li, Xue Li, Weijie Gao, Hyun-Chul Jeong

**Affiliations:** 1 Department of Physical Education, Jeonbuk National University, Jeonju, Republic of Korea; 2 College of Physical Education, Northeast Electric Power University, Jilin, China

**Keywords:** balance, basketball, core muscle activation, instability resistance training, performance stability

## Abstract

**Background:**

Basketball requires players to perform complex technical actions under conditions of dynamic instability. Traditional stability and strength training mainly improves muscle strength; however, it is often insufficient for improving postural control and neuromuscular coordination. Instability resistance training (IRT) enhances sensory integration, core activation, and dynamic stability by introducing unstable supporting surfaces and disturbing environments.

**Objectives:**

Based on sensory–motor control theory, core stability theory, and dynamic system theory, this study investigated the effects of IRT on static balance, dynamic balance, core muscle recruitment, and performance during a controlled stop-jump shooting task in college basketball players.

**Methods:**

Sixty college basketball players were randomly divided into two groups: an IRT and a traditional strength training group (TST). The intervention lasted for 8 weeks, with training sessions conducted twice weekly for 90 min each. Outcome measures included the modified Romberg test, the Y-Balance test, core muscle activation expressed as a percentage of maximum voluntary contraction (%MVC) using surface electromyography, and performance in a controlled stop-jump shooting task. Data were analyzed using paired-sample and independent-sample *t*-tests, with the significance level set at *p* < 0.05. Effect sizes were calculated using Cohen's *d* formula, where |*d*| < 0.2 indicated a minor effect, 0.2 ≤ |*d*| < 0.5 indicated a small effect, 0.5 ≤ |*d*| < 0.8 indicated a moderate effect, and |*d*| ≥ 0.8 indicated a large effect; 95% confidence intervals were calculated based on the *t*-distribution.

**Results:**

After training, the IRT group demonstrated significant improvements in static balance (+7.14 s, *p* < 0.001, |*d*| = 0.630), dynamic balance in three directions (+6.39%–9.54%, *p* < 0.001, |*d*| 0.446–0.543), and core muscle recruitment intensity (transversus abdominis, erector spinae, and multifidus +5.79% vs. 6.72% MVC, *p* < 0.001, |*d*| = 0.784–0.872). The IRT group also performed significantly better than the TST group in the controlled stop-jump shooting task and in the fatigue-related late-set shooting indicator (*p* < 0.05, |*d*| = 0.339, 0.255).

**Conclusion:**

IRT achieves multilevel neuromuscular adaptation by enhancing sensory integration, improving core stability, and promoting motor coordination and self-organization. Compared with traditional strength training, IRT appears to be more effective in developing functional strength and dynamic stability ability of basketball players. Future studies should expand the sample size, group participants according to field position, and include electromyographic timing and spectral indicators to further clarify the neuromuscular mechanisms underlying core control.

## 1 Introduction

Basketball is a high-intensity intermittent team sport that requires players to repeatedly perform accelerations, decelerations, rapid changes of direction, jumps, and technically demanding actions under time pressure and contact from opponents (1). These movements require high requirements for body balance, core control, and neuromuscular coordination (2).

Traditional strength training (TST) generally involves high-load resistance training performed on stable support surfaces, with the aim of enhancing muscle strength and power (3, 4). However, the competitive environment itself is full of uncertainties and disturbances. Athletes should not only generate force but also stabilize the postures and control movement directions when they are in an unstable environment (5). However, because many basketball actions are executed under rapidly changing and mechanically challenging conditions, stable-surface strength training alone may not be sufficient to address the demands of postural control, balance adaptation, and trunk stabilization during sport-specific movement (2, 5–7).

A training method involving the purposeful application of unstable support surfaces or external perturbations to promote the coordinated action of peripheral and trunk-stabilizing muscles is called instability resistance training (IRT) (5, 7, 8). This method aims to increase the proprioception, postural control, and neuromuscular control of athletes (8). Compared with conventional training on stable activities, IRT focuses on neural regulation and body coordination rather than solely on force production (9). In the present study, IRT was interpreted through three complementary perspectives: sensorimotor control theory, core stability theory, and dynamic systems theory (10–12).

According to sensorimotor control theory, the perceived stability and efficiency of motor activity are related to the overall control of visual, vestibular, and proprioceptive signals (13). When athletes participate in instability resistance exercise, the nervous system receives continuous postural perturbations that compel the system to constantly reorganize sensory input from joints, muscles, and the skin, thereby improving adaptability to balance perturbations and speed of postural loading (14). Continuous sensorimotor stimulation facilitates neural plasticity process and enhances the responsiveness and coordination of the central nervous system (7, 15).

Core stability theory is based on the idea that the core musculature, encompassing the transversus abdominis (TrA), multifidus, pelvic floor muscles, and erector spinae (ES), is important in maintaining body posture and facilitating force transmission (10). When the support is in a fluctuating state, the core muscles must contract in a synchronized pattern to maintain body stability, consequently enhancing the efficacy of force transfer between the core and the limbs. It has been established that, in athletes and endurance-trained athletes, core training demonstrated in the form of long-term instability can substantially improve postural control and lower-limb stability (16).

According to dynamic systems theory, motor behavior arises from the interaction among individual, task, and environmental constraints (17). Instability resistance training changes the environmental constraints, thereby motivating individuals to develop the best coordination patterns of movements within a new dynamic balance (11, 12). Such a process aligns with the concept of non-linear learning and increases self-regulation and adaptability of athletes in challenging settings.

Overall, the effect of instability resistance training in athletes can be summarized as follows: creating unstable conditions → enhancing neuromuscular control → optimizing core stability and postural regulation → improving task-specific motor performance under controlled testing conditions. Through this process, athletes will be able to restore balance faster, improve stability in competition control (technical), and reduce movement distortion and the risk of injury during physical contacts.

Therefore, from a theoretical perspective, instability resistance training is not only a functional supplement to traditional strength training but also a key approach for developing dynamic stability and functional strength among basketball players (5, 18).

Based on the three aforementioned theories, this study established a “theory–mechanism–variable” logical framework (see Table 1), which clarifies the corresponding mechanisms and measurement indicators of each theoretical framework in the experimental design, providing a theoretical basis for subsequent hypothesis testing (10–12).

**Table 1:** Theory–mechanism–variable logical chain structure.

Theoretical framework	Core mechanism	Corresponding experimental variable	Testing hypothesis
Sensorimotor control theory	Sensory integration and postural regulation	Static balance (modified Romberg test)	Instability resistance training significantly enhances static balance performance
		TrA muscle	Instability resistance training significantly enhances TrA recruitment intensity and is positively correlated with improved static balance
		MVC (abdominal hollowing)	
Core stability theory	Core activation and neural fatigue resistance	Dynamic balance (Y-Balance), fatigue-related late-set stop-jump shooting task performance	Instability resistance training markedly improves dynamic balance and fatigue-related stop-jump shooting task performance
		Erector spinae, multifidus MVC (prone isometric back extension, quadruped single-leg back extension)	Instability resistance training significantly enhances recruitment intensity of the erector spinae and multifidus muscles and is positively correlated with dynamic balance and fatigue-related task performance
Dynamic systems theory	Environmental perturbations trigger movement self-organization	Integrated task outcomes (balance measures and controlled stop-jump shooting task performance)	Instability resistance training promotes multivariable synergistic optimization
		Mean MVC of the TrA, erector spinae, and multifidus muscles	Instability resistance training significantly enhances the overall recruitment intensity of core muscle groups and contributes to improvements in the measured balance and task-specific shooting outcomes

Based on the above theoretical framework, this study designed an experimental protocol centered on static and dynamic balance, core muscle activation, and controlled stop-jump shooting task performance as the core variables. The detailed design is presented in the following section.

## 2 Method

### 2.1 Research subjects

A total of 60 basketball players from a university basketball team in China were recruited and randomly assigned to either the IRT group or the TST group, with 30 participants in each group. Table 2 presents the baseline assessment results. Independent-samples *t*-tests showed no significant differences between the two groups in age, years of basketball training experience, height, or body weight (all *p*’s > 0.05). This study was conducted during normal basketball training; the training intervention consisted of conventional physical training methods that did not pose additional physical or psychological risks to participants. All participants signed informed consent forms. G*Power 3.1 was used in this study to perform *a priori* sample size estimation. The analysis was based on a two-sided independent-samples *t*-test, test, with the effect size set at *d* = 0.80, significance level *α* = 0.05, statistical power 1 − *β* = 0.80, and a distribution ratio of the IRT group to the TST group of 1:1. The calculation showed that the study needs a minimum of 52 subjects, with 26 participants in each group. Considering the possibility of sample dropout during the training intervention, this study plans to recruit 30 participants per group, for a total sample size of 60.

**Table 2:** Baseline characteristics of the participants.

Variable	IRT (*n* = 30)	TST (*n* = 30)	*T*	*P*
Age (years)	20.83 ± 1.90	20.67 ± 0.99	0.427	0.671
Basketball training experience (years)	5.17 ± 1.02	5.30 ± 0.99	−0.514	0.609
Height (cm)	184.86 ± 9.34	183.12 ± 9.01	0.735	0.466
Body mass (kg)	78.64 ± 11.47	76.32 ± 10.97	0.800	0.427

*P* > 0.05 indicates no significant difference; *P < 0.05 indicates a significant difference; **P < 0.01 indicates a highly significant difference; ***P < 0.001 indicates an extremely significant difference.

### 2.2 Experimental methods

**Figure 1:**
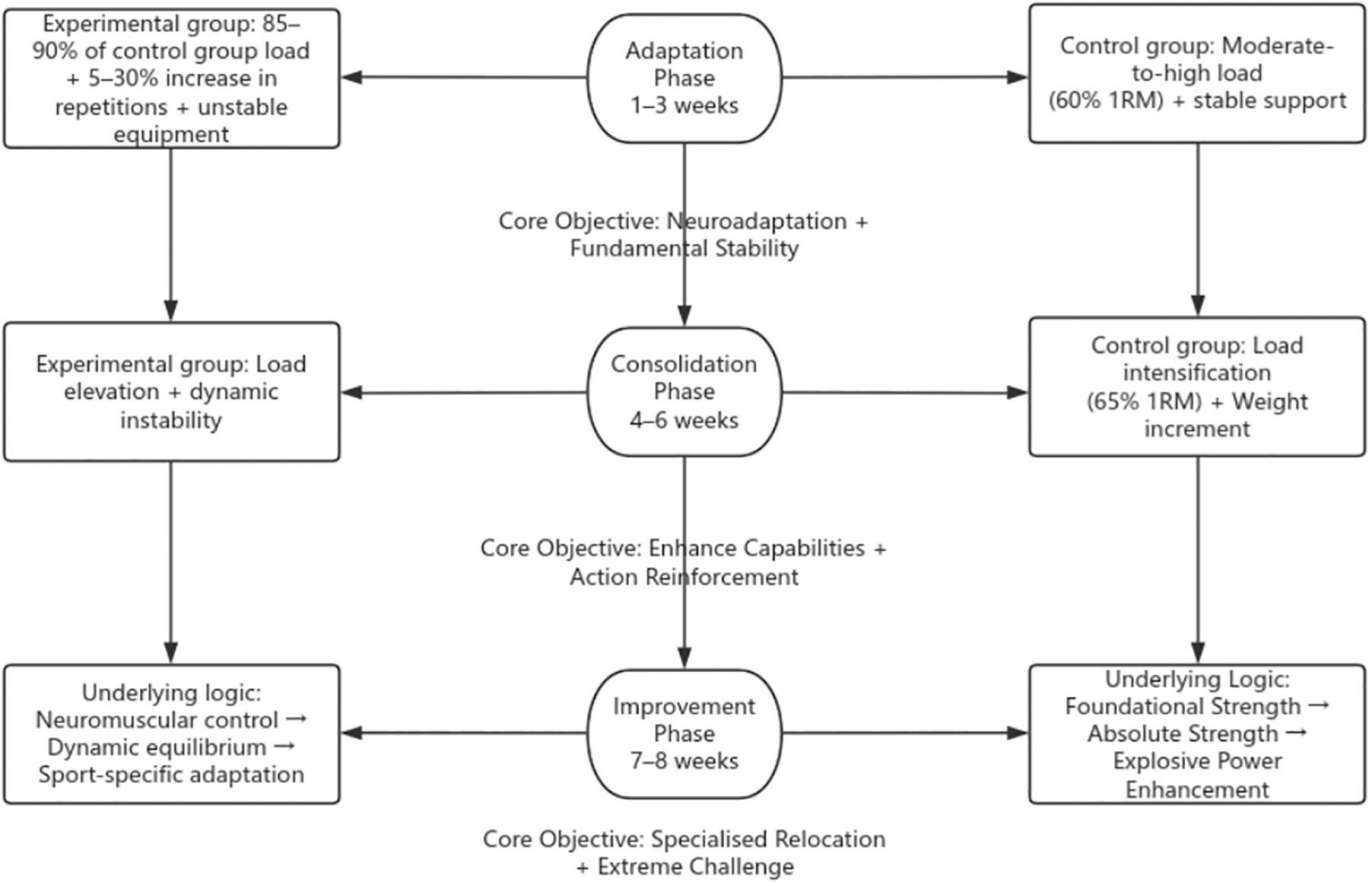
Conceptual diagram of progressive stages.

During the 8-week training period, the IRT group performed instability resistance training twice weekly, on Tuesday and Thursday afternoons, with each session lasting 90 min. The TST group followed their original training plan and participated in regular basketball strength training at the corresponding time points. The experimental design followed the progressive stage concept illustrated in Figure 1. Training load was designed according to the principle of “absolute load matching +  training volume compensation.” Specific training content is detailed in Table 3. Training logs were meticulously recorded, and conditions of athletes were monitored to ensure consistency in supplementary training content, load volume, and training duration across participants.

**Table 3:** Training content.

Stage	Training area	IRT group training content	TST group training content
1–3 weeks adaptation phase	Upper limbs	Seated press on a Swiss ball	Seated press
		(51% 1RM, 3 × 14)	(60% 1RM, 3 × 12)
		Supine press on a Swiss ball	Bench Press
		(51% 1RM, 3 × 14)	(60% 1RM, 3 × 12)
		Push-ups on a balance pad	Push-ups
		(85% 1BW, 3 × 15)	(100% BW, 3 × 10)
	Lower limbs	Wall-supported static squat on a Swiss ball	Wall squats
		(85% BW, 3 × 52s)	(100% BW, 3 × 45s)
		Single-leg calf raises on a balance pad	Single-leg calf raises
		(85%BW, 3 × 18)	(100% BW + 2kg, 3 × 15)
	Core	Plank on a balance pad	Plank
		(100%BW, 3 × 66s)	(100% BW, 3 × 60s)
		Side-lying foot support on a Swiss ball	Side plank
		(80%BW, 3 × 16/side)	(100% BW, 3 × 15/side)
		Static back extension on a Swiss ball	Static back extension
		(75%BW, 3 × 44s)	(100% BW, 3 × 40s)
4–6 weeks consolidation phase	Upper limbs	Prone dumbbell press-ups on a Swiss ball	Prone barbell curl
		(55%1RM, 3 × 11)	(65% 1RM, 3 × 10)
		Prone dumbbell lifts on a Swiss ball	Prone kettlebell deadlift
		(55% 1RM, 3 × 11)	(65% 1RM, 3 × 10)
		Prone push-ups on a Swiss ball	Push-ups
		(75% BW, 3 × 13)	(100% BW + 5kg, 3 × 12)
	Lower limbs	Half-squat jumps on a Bosu ball	Half squat jumps
		(90% BW, 3 × 11)	(50% 1RM, 3 × 10)
		Bulgarian split squats on a balance pad	Bulgarian split squats
		(51% 1RM, 3 × 9/side)	(60% 1RM, 3 × 8/side)
	Core	Balance pad supine shoulder press with hip thrust	Supine shoulder press with hip thrust
		(35% 1RM, 3 × 14)	(40% 1RM, 3 × 12)
		Swiss ball side-lying hip thrust	Side-lying hip thrust
		(85% BW, 3 × 13/side)	(100%BW + 3kg, 3 × 15/side)
		Swiss ball prone back raise	Prone back raise
		(75% BW, 3 × 16)	(30%1RM, 3 × 15)
7–8 weeks Improvement phase	Upper limbs	Swiss ball supine press	Supine press
		(68% 1RM, 3 × 9)	(75%1RM, 3 × 8)
		Swiss ball supine lateral raises	Supine lateral raise
		(54% 1RM, 3 × 9/side)	(60%1RM, 3 × 8/side)
		Swiss ball weighted push-ups	Weighted push-up
		(100% BW + 11kg, 3 × 11)	(100%BW + 12kg, 3 × 10)
	Lower limbs	Medicine ball weighted squats	Barbell squat
		(51% 1RM, 3 × 9)	(70% 1RM, 3 × 8)
		Medicine ball weighted half-squat jumps	Weighted half-squat jumps
		(51% 1RM, 3 × 9)	(60% 1RM, 3 × 8)
	Core	Swiss ball weighted prone leg raises	Weighted prone leg raises
		(35% 1RM, 3 × 11)	(40% 1RM, 3 × 10)
		Swiss ball weighted side-lying leg raises	Weighted side-lying leg raises
		(30% 1RM, 3 × 11/side)	(35% 1RM, 3 × 10/side)
		Swiss ball weighted supine leg raises	Weighted supine leg raises
		(40% 1RM, 3 × 13)	(45% 1RM, 3 × 12)

1RM, one repetition maximum; BW, body weight.

### 2.3 Measurement methods

Before and after the experiment, each athlete in both the IRT and TST groups was tested for static and dynamic balance ability, core muscle activation, and performance on a controlled stop-jump shooting task. The selected assessment indicators included the modified Romberg test, the Y-Balance test, surface electromyographic (EMG) signals of core muscles, and the stop-jump shooting test.

#### 2.3.1 Modified Romberg test method

Participants were required to maintain balance in a tandem stance, placing the toe of the back foot directly against the heel of the front foot on the same straight line. Timing began once the participant closed their eyes and ended when noticeable body sway occurred. Each participant completed three trials, and the best performance was recorded (19, 20).

#### 2.3.2 Y-Balance test method

Participants stood barefoot at the center of a Y-shaped testing apparatus, with one supporting leg on the ground and the other extended as far as possible in three directions as good as possible: anterior (ANT), posteromedial (PM), and posterolateral (PL). In each directional test, the participants were instructed to uphold the supporting leg, maintain a natural upright position of the trunk, lightly touch the end of the reach indicator with the non-supporting leg before returning to the starting position, place both hands on hips, and avoid any compensatory movements. All directions were run thrice, with a 3-min rest period between runs to reduce fatigue. On the left supporting leg, the test was performed, after which the participants rested for 5 min, and then the test on the right leg was performed. The assessment involved the ratio of the maximum valid reaching distance in each direction to the leg length (between the anterior superior iliac spine and the medial malleolus) (21).

#### 2.3.3 Measurement method of core muscle electromyographic signals

The level of core muscle activation during unstable movements was analyzed using surface electromyography (sEMG). In this experiment, the target muscles to be measured were identified as TrA, ES, and multifidus (MF). Bipolar Ag/AgCl electrodes were placed with an interelectrode distance of approximately 2 cm and aligned parallel to the direction of muscle fibers: electrodes for the TrA were positioned 2–3 cm below the umbilicus, slightly medially and inferiorly; electrodes for the ES were placed 2 cm lateral to the spinous process of the third lumbar vertebra (L3); electrodes for the MF were positioned 2–3 cm lateral to the spinous process of the fifth lumbar vertebra (L5). To obtain maximum voluntary contraction (MVC) values, each muscle group performed three maximal isometric contractions, each held for 5 s, with a 60-s interval between contractions (22–24, 42). The root mean square (RMS) value of the middle 1 s was calculated for each contraction, and the highest RMS value was selected as the MVC reference value for the muscle group. The experiment protocol consisted of three exercises: abdominal hollowing, prone isometric back extension, and quadruped single-leg back extension. Every movement was performed three times, and each repetition lasted 5 s, with a break of 3 min between repeats. After full-wave rectification and smoothing (6-Hz low-pass filtering) of the signals, the RMS value was calculated. The average RMS value of the middle 1-s segment was used as the mean measured RMS value. Finally, the muscle activation level during each movement was expressed as %MVC, calculated using the following formula: %MVC = (mean measured RMS value/MVC reference value) × 100%.

#### 2.3.4 Controlled stop-jump shooting task

In the stop-jump shooting task, athletes began dribbling from the backcourt baseline, accelerated forward the frontcourt, and advanced to the free-throw line position, where a simulated defender prop was placed with its arms raised upward and positioned on the side of the non-ball-holding hand of the athlete; when the athlete advanced to the marked point located 0.5 m away from the simulated defender, they immediately executed a stop-jump shot, after which they quickly ran back to the backcourt baseline, stepped on the baseline, immediately received the ball, and repeated the aforementioned movement. Each set consisted of 20 stop-jump shots, and a total of three sets were tested; the set with the highest shooting percentage was recorded, and the shooting percentage of the last 10 shots within that set was documented as a fatigue-related task performance indicator.

### 2.4 Data processing

SPSS 29.0 was used for statistical processing and analysis of experimental indicators, with the significance level set at *p* < 0.05. Effect sizes were calculated using Cohen's *d* formula, where |*d*| < 0.2 indicates a minor effect, 0.2 ≤ |*d*| < 0.5 indicates a small effect, 0.5 ≤ |*d*| < 0.8 indicates a moderate effect, and |*d*| ≥ 0.8 indicates a large effect. In addition, 95% confidence intervals were calculated based on the *t*-distribution.

## 3 Results

### 3.1 Static balance

The modified Romberg test was based on the accuracy of the eyes-closed tandem stance duration as the main parameter to assess the ability of the participants in their static balance control and proprioceptive integration. Table 4 provides the results.

**Table 4:** Static balancing capability.

Group	Measurement phase	Mean	Standard deviation	Mean difference in change	Within-group effect size (*d*, 95% CI)	Intragroup comparison (*t*, *p*)	Intergroup comparison (pretest *p*, posttest *p*)	Between-group effect size (*d*, 95% CI)
IRT group	Pretest	23.78	5.27	+7.14	−0.630 [−0.992, −0.269]	*t* = −27.73	Pretest *p* = 0.357	1.001 (0.528,1.467)
	Posttest	30.92	6.07			*p* < 0.001***		
TST group	Pretest	25.05	5.26	+0.18	−0.034 [−0.356, 0.288]	*t* = −3.29	Posttest *p* < 0.001***	
	Posttest	25.23	5.23			*p* = 0.003**		

*P* > 0.05 indicates no significant difference; *P < 0.05 indicates a significant difference; **P < 0.01 indicates a highly significant difference; ***P < 0.001 indicates an extremely significant difference.

Among the within-group training impacts, the IRT and TST groups showed a very significant difference. The mean time difference in the IRT group was +7.14 s [95% CI (−0.992, −0.269), |*d*| = 0.630, *p* < 0.001], which indicated that instability resistance training had a significant effect on enhancing the ability to balance in a static position of participants. The mean change in the TST group was +0.18 s [95% CI (−0.356, 0.288), |*d*| = 0.034, *p* = 0.003]. The extremely small mean difference and negligible effect size demonstrate that, although the change was statistically significant, the improvement was minimal.

Based on the between-group comparison, the pretraining outcome showed *p* = 0.357 > 0.05, indicating no significant difference in static balance ability between the two groups, which negates the effect of initial differences in the baseline on the experimental outcome. The mean value of the IRT group was significantly higher than that of the TST group after training, with *p* < 0.001 [95% CI (0.528, 1.467), |*d*| = 1.001], and this is additional evidence that instability resistance training has a definite advantage in enhancing static balance ability compared to traditional strength training.

### 3.2 Dynamic balance

The Y-Balance test was used to assess the participants’ multidirectional dynamic balance and ability to maintain limb control using the ratio of the effective reach distance in the three directions, namely, ANT, PM, and PL, to leg length. Table 5 lists the results of the provided sample.

**Table 5:** Dynamic balancing capability.

Group	Direction of measurement	Measurement phase	Mean	Standard deviation	Mean difference in change	Within-group effect size (*d*, 95% CI)	Intragroup comparison (*t*, *p*)	Intergroup comparison (pretest *p*, posttest *p*)	Between-group effect size (*d*, 95% CI)
IRT group	ANT	Pretest	101.25	8.16	+6.39	−0.446 [−0.808, −0.084]	*t* = −54.90	Pretest *p* = 0.973	0.778 (0.315, 1.234)
		Posttest	107.64	7.76			*p* < 0.001***		
TST group	ANT	Pretest	101.31	6.84	+0.51	−0.075 [−0.397, 0.247]	*t* = −1.80	Posttest *p* = 0.004**	
		Posttest	101.82	7.20			*p* = 0.083		
IRT group	PM	Pretest	96.97	8.72	+9.54	−0.534 [−0.896, −0.173]	*t* = −42.58	Pretest *p* = 0.560	0.962 (0.493, 1.424)
		Posttest	106.51	8.50			*p* < 0.001***		
TST group	PM	Pretest	98.27	8.36	+0.04	−0.005 [−0.327, 0.317]	*t* = −0.28	Posttest *p* < 0.001***	
		Posttest	98.31	8.56			*p* = −780		
IRT group	PL	Pretest	96.04	8.50	+9.46	−0.543 [−0.904, −0.181]	*t* = −44.52	Pretest *p* = 0.861	1.090 (0.615, 1.557)
		Posttest	105.50	8.09			*p* < 0.001***		
TST group	PL	Pretest	96.42	8.24	+0.12	−0.015 [−0.337, 0.307]	*t* = −0.90	Posttest *p* < 0.001***	
		Posttest	96.54	8.34			*p* = 0.378		

ANT, anterior; PM, posteromedial; PL, posterolateral. *P* > 0.05 indicates no significant difference; *P < 0.05 indicates a significant difference; **P < 0.01 indicates a highly significant difference; ***P < 0.001 indicates an extremely significant difference.

Based on within-group training effects, the IRT group showed a significant increase in dynamic balance in all three directions. Specifically, in the ANT, PM, and PL directions, the mean differences in change were +6.39% (95% CI [−0.808, −0.084], |*d*| = 0.446), +9.54% (95% CI [−0.896, −0.173], |*d*| = 0.534), and +9.46% (95% CI [−0.904, −0.181], |*d*| = 0.543), respectively, with all *p*’s < 0.001. In contrast, no significant changes were observed in the TST group in any of the three directions, with all *p* values > 0.05.

Intergroup comparisons showed no significant differences between the IRT and TST groups prior to training in the ANT, PM, and PL directions (*p* = 0.973, 0.560, and 0.861, respectively; all *p*’s > 0.05), indicating randomization and fairness of the IRT grouping. Following the training, the IRT group demonstrated much higher ratios in the three directions than the TST group: ANT: *p* = 0.004 < 0.05, 95% CI (0.315, 1.234), |*d*| = 0.778; PM: *p* < 0.001, 95% CI (0.493, 1.424), |*d*| = 0.962; and PL: *p* < 0.001, 95% CI (0.615, 1.557), |*d*| = 1.090, indicating that the IRT group achieved greater improvements across all directions than the TST group. These findings indicate that 8-week instability resistance training program showed a significantly larger effect on enhancing dynamic balance ability than traditional strength training, with this effect being significantly more pronounced in the PM and PL directions, which involve greater neuromuscular control requirements.

### 3.3 Core muscle activation

sEMG testing using the percentage of maximum voluntary contraction (%MVC) as an indicator was employed to measure the capacity of the muscle to recruitment, and Table 6 presents the result of the test.

**Table 6:** Core muscle activation.

Group	Measurement indicators	Measurement phase	Mean	Standard deviation	Mean difference in change	Within-group effect size (*d*, 95% CI)	Intragroup comparison (*t*, *p*)	Intergroup comparison (pretest *p*, posttest *p*)	Between-group effect size (*d*, 95% CI)
IRT group	TrA	Pretest	29.42	4.02	+6.72	−0.802 [−1.163, −0.440]	*t* = −35.73	Pretest *p* = 0.397	1.564 (1.038, 2.075)
		Posttest	36.14	3.83			*p* < 0.001***		
TST group	TrA	Pretest	30.26	3.57	+0.11	−0.031 [−0.353, 0.291]	*t* = −2.45	Posttest *p* < 0.001***	
		Posttest	30.37	3.53			*p* = 0.021*		
IRT group	ES	Pretest	26.14	3.06	+5.79	−0.784 [−1.146, −0.423]	*t* = −26.84	Pretest *p* = 0.933	1.599 (1.068, 2.113)
		Posttest	31.92	3.97			*p* < 0.001***		
TST group	ES	Pretest	26.20	2.84	+0.13	−0.046 [−0.368, 0.276]	*t* = −2.66	Posttest *p* < 0.001***	
		Posttest	26.33	2.90			*p* = 0.013*		
IRT group	MF	Pretest	25.68	2.98	+6.67	−0.872 [−1.233, −0.510]	*t* = −40.05	Pretest *p* = 0.768	1.867 (1.298, 2.416)
		Posttest	32.35	3.67			*p* < 0.001***		
TST group	MF	Pretest	25.91	3.01	+0.11	−0.037 [−0.359, 0.285]	*t* = −2.59	Posttest *p* < 0.001***	
		Posttest	26.02	3.09			*p* = 0.015*		

TrA, transversus abdominis; ES,  erector spinae; MF, multifidus. *P* > 0.05 indicates no significant difference; *P < 0.05 indicates a significant difference; **P < 0.01 indicates a highly significant difference; ***P < 0.001 indicates an extremely significant difference.

Based on the within-group comparison, EMG activation levels in the IRT group for the TrA, ES, and MF were significantly improved after 8 weeks of instability resistance training. In the IRT group, the mean difference in change in activation levels of TrA, ES, and MF increased by +6.72, +5.79, and +6.67%MVC, respectively, and the corresponding |*d*| values were 0.802, 0.784, and 0.872, with all *p*’s < 0.001. In the TST group, all three indicators showed only minor, but statistically significant, changes, with all 0.05 < *p* < 0.01 and all |*d*|’s < 0.2. These results indicate that instability resistance training is more effective than traditional stable surface training for improving core muscle activation.

Intergroup comparisons revealed no value differences between the IRT and the TST groups prior to training in the %MVC values of the TrA, ES, and MF (*p* = 0.397, 0.993, and 0.768, respectively, all *p*’s > 0.05), indicating the randomization and fairness of the IRT grouping. Following training, the IRT group recorded significantly greater %MVC values in all three muscles compared with the TST group, and the difference was significant (all *p*’s < 0.001, and all |*d*|’s > 0.8), which demonstrates that instability resistance training was able to enhance the level of core muscle activation significantly, although the TrA and multifidus were affected more effectively.

### 3.4 Controlled stop-jump shooting task performance

The stop-jump shooting test was used as a controlled task-specific assessment. The total number of successful shots out of 20 attempts was used as an indicator of stop-jump shooting task accuracy, whereas the number of successful shots in the final 10 attempts of the best-performing set was used as a fatigue-related task performance indicator. The results are presented in Table 7.

**Table 7:** Outcomes of the controlled stop-jump shooting task.

Group	Measurement indicators	Measurement phase	Mean	Standard deviation	Mean difference in change	Within-group effect size (*d*, 95% CI)	Intragroup comparison (*t*, *p*)	Intergroup comparison (pretest *p*, posttest *p*)	Between-group effect size (*d*, 95% CI)
IRT group	Stop-jump shooting task accuracy	Pretest	8.77	2.81	+2.33	−0.339 [−0.700, 0.023]	*t* = −13.33	Pretest *p* = 0.443	0.652 (0.195, 1.102)
		Posttest	11.10	3.17			*p* < 0.001***		
TST group	Stop-jump shooting task accuracy	Pretest	9.33	2.87	−0.26	0.092 [−0.229, 0.413]	*t* = 1.39	Posttest *p* = 0.014*	
		Posttest	9.07	3.06			*p* = 0.174		
IRT group	Fatigue-related late-set shooting accuracy	Pretest	2.57	1.65	+1.23	−0.255 [−0.617, 0.106]	*t* = −9.28	Pretest *p* = 0.639	0.950 (0.482, 1.411)
		Posttest	3.80	1.94			*p* < 0.001***		
TST group	Fatigue-related late-set shooting accuracy	Pretest	2.37	1.63	−0.30	0.186 [−0.136, 0.508]	*t* = 1.87	Posttest *p* = 0.001***	
		Posttest	2.07	1.70			*p* = 0.071		

*P* > 0.05 indicates no significant difference; *P < 0.05 indicates a significant difference; **P < 0.01 indicates a highly significant difference; ***P < 0.001 indicates an extremely significant difference.

Within-group comparisons revealed that the IRT group showed a significant change in performance in the controlled stop-jump shooting task. In the IRT group, the mean difference in change was +2.33 [*p* < 0.001, 95% CI (−0.700, 0.023), |*d*| = 0.339], which showed that instability resistance training can significantly improve task performance under the standardized test protocol. In the TST group, the mean difference in change was −0.26.[*p* = 0.174 > 0.05, 95% CI (−0.229, 0.413), |*d*| = 0.092], which indicated that traditional strength training has no significant effect on task performance under the standardized test protocol.

The IRT group also showed a significant improvement in the fatigue-related late-set shooting indicator [*p* < 0.001, 95% CI (−0.617, 0.106), |*d*| = 0.255], whereas no significant difference was observed in the TST group [*p* > 0.05, 95% CI (−0.136, 0.508), |*d*| = 0.186]. These findings suggest that instability resistance training may help athletes maintain task performance during the later phase of repeated shooting under the present testing conditions.

Between-group comparisons revealed no significant differences in either stop-jump shooting task accuracy (*p* = 0.443) or fatigue-related late-set shooting accuracy (*p* = 0.639). Following training, however, the IRT group demonstrated significantly higher scores than the TST group on both performance measures, with *p* = 0.014 < 0.05, 95% CI (0.195, 1.102), |*d*| = 0.652 and *p* = 0.001 < 0.01, 95% CI (0.482, 1.411), |*d*| = 0.950, respectively. These findings should be interpreted as task-specific improvements under the present assessment conditions rather than as direct evidence of overall in-game shooting performance.

## 4 Discussion

**Figure 2:**
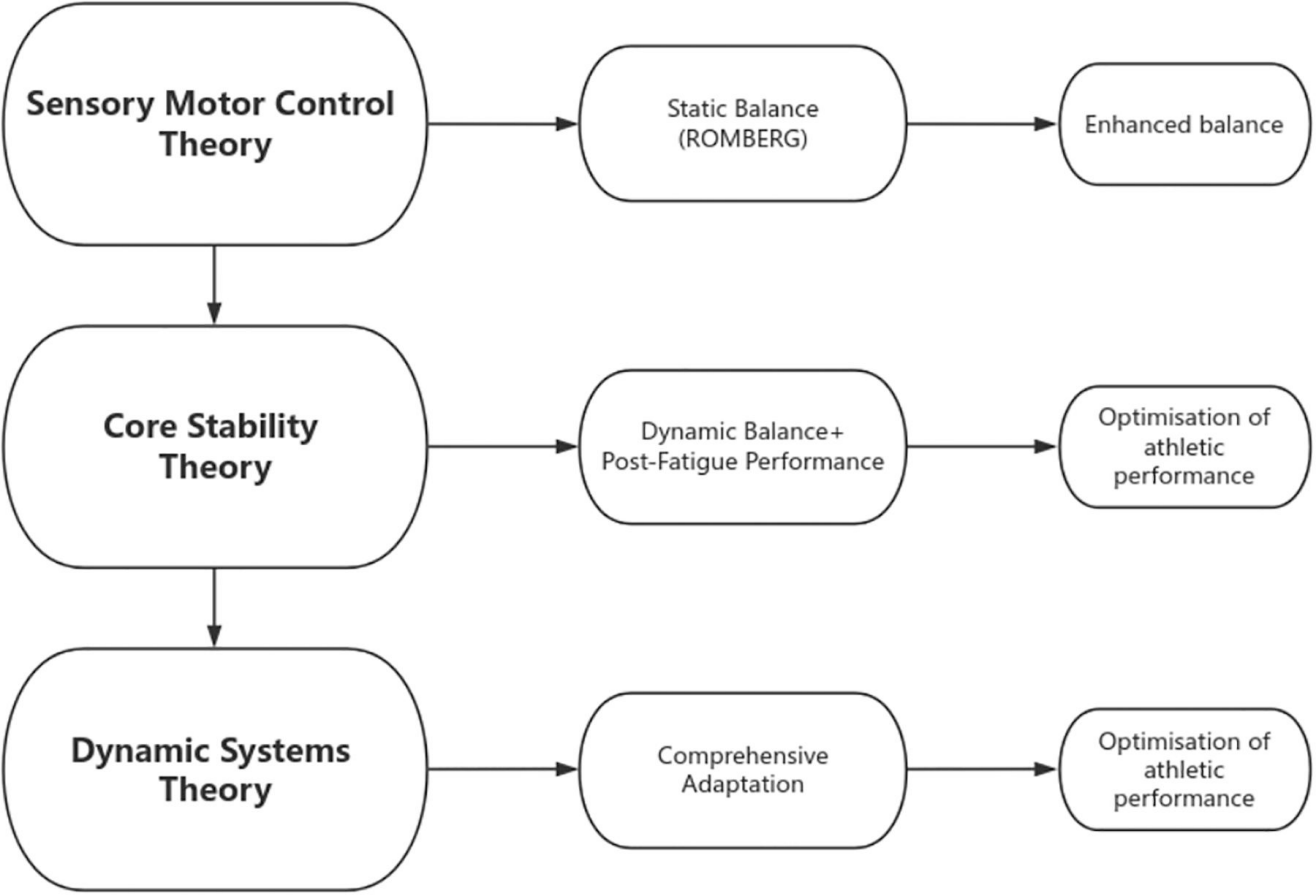
Integrated model of the mechanism of action underlying instability resistance training.

According to the analysis of the above results, this study integrated the examination of the action pathway of instability resistance training, as depicted in Figure 2, which illustrates the flow of connections from the theoretical mechanism to the experimental behavior of the system and plays a leading role among the three theoretical frameworks of sensorimotor control, core stability, and dynamic systems. In addition, the surface electromyography (%MVC) of core muscles serves as the main physiological evidence for integrating the “theory–mechanism–behavioral performance” into the closed-loop logical circle.

The present research was based on sensorimotor control theory, core stability theory, and dynamic systems theory to investigate the effects of instability resistance training on balance ability and controlled stop-jump shooting task performance in college basketball players. To confirm the relevance of these three theoretical models in a sports training scenario, the experiment involved an integrated analysis and theoretical interpretation of the outcomes, grounded in four experimental variables, namely, static balance, dynamic balance, core muscle recruitment, and controlled stop-jump shooting task performance.

### 4.1 Sensorimotor control theory perspective: instability resistance training promotes sensory integration and improves static balance

According to the sensorimotor control theory, motor performance stability is achieved through the concerted functioning of the visual, vestibular, and proprioceptive systems (13). The modified Romberg test is performed with the participant’s eyes closed and is used to assess the ability of an individual to stand on their feet stabilized in one position to provide a measure of the efficiency of vestibular and proprioceptive integration. It was also observed that the results of this experiment demonstrated that the IRT group [the static balance time improved by +7.14 s, 95% CI (−0.992, −0.269), |*d*| = 0.630, *p* < 0.001] and the TST group (the static balance time improved by +0.18 s, 95% CI (−0.356, −0.288), |*d*| = 0.034, *p* = 0.003) showed significant differences after 8 weeks of intervention.

Such a significant difference shows that the instability resistance training process strengthens feedback regulation of the vestibular system and proprioceptors (e.g., muscle spindles) through persistent postural disturbances (15), thereby causing the nervous system to continuously restructure sensory inputs and enhance the accuracy and speed of postural adaptation (25). This mechanism was further verified by the surface electromyography outcomes of the core muscles. Data from the IRT group showed a significant increase in the activation level of the TrA muscle. Being a deep core muscle responsible for maintaining balance in a free body upright position, increased TrA activation was associated with a longer time of stability. This observation suggests that sensorimotor integration may contribute not only to neural regulation but also to more precise core muscle recruitment and enhanced postural control. These adaptations provide a plausible physiological explanation for the improvement in static balance performance observed after training.

### 4.2 Core stability theory perspective: core control enhances dynamic balance and sports-specific postfatigue performance

According to core stability theory, the core musculature, including TrA, multifidus, and pelvic floor muscles, plays a key role in maintaining posture and facilitating force transmission (10). Both the Y-Balance test and the stop-jump shooting task with a sudden stop entail coordinated actions of the lower limbs on the core. In the meantime, electromyographic data from the core muscles serve as one of the links between the core activation mechanism and performance at the behavioral level.

The outcomes of this experiment revealed that the IRT group exhibited remarkable progress in dynamic balance ratios in all three directions (mean progressive growth of +6.39%, *p* < 0.001, |*d*| values of 0.446, 0.534, and 0.543, respectively), but the improvement was greater in the PM and PL directions. The two directions imply well-coordinated hip abduction, internal rotation, and ankle plantar flexion, aided by neuromuscular control of the core and hip-stabilizing muscles (26). The electromyographic data also provided further explanation of how this particular behavioral improvement had been achieved. The activation levels of the ES and MF muscles increased significantly. The ES assists in spinal longitudinal stability, whereas the MF controls small details of intersegmental balance. The concomitant improvement in their recruitment can help participants control center-of-mass changes more precisely during dynamic reach tasks, decreasing compensatory motions and leading to considerable improvements in PM and PL balance performance. Thus, instability resistance training using Swiss and BOSU balls is effective in increasing the capacity of the core and lower limbs to synergistically contract, and participants can obtain better postural control when extending dynamically (27).

In terms of fatigue-related late-set shooting performance, the IRT group indicated an increase of 1.23 successful shots [*p* < 0.001, 95% CI (−0.617, 0.106), |*d*| = 0.255] in the final 10 stop-jump shooting attempts, a figure that was directly linked to the recruitment nature of core muscles. Participants who underwent instability resistance training were able to maintain balance during repeated postural stabilization tasks, which not only improved baseline recruitment strength of the core muscles but also enhanced neural control of fatigue resistance. The core muscles were capable of maintaining %MVC at the same level, regardless of high-intensity repetitive movements, preventing trunk flexion and high-body instability that may upset the shooting position.

On the other hand, core muscle recruitment strength increased modestly in the TST group (0.11%–0.13%MVC, all *p*’s < 0.05, all |*d*| < 0.2). This small enhancement of activation was not sufficient to sustain postures during fatigue, resulting in no significant difference in postfatigue performance. This comparison also indicates a core theory of stability, an assumption about the assemblage of core strength, postural control, and movement stability. It shows that instability resistance training increases endurance rate and neuromuscular fatigue resistance of the core muscles (18, 28). Further, it implies that the muscle recruitment capacity of the core muscles is a critical process that contributes to increased stability during critical movements in basketball, which follow high-intensity physical confrontation.

### 4.3 Dynamic systems theory perspective: environmental perturbations facilitate movement self-organization and integrated motor adaptation

Dynamic systems theory proposes that movement patterns emerge from the interaction among individual, task, and environmental constraints (17). By artificially altering the support surface, the center of gravity perturbations, and load uncertainty, instability resistance training has the probable effect of changing environmental constraints, compelling athletes to self-organize more efficient movement coordination structures under dynamic conditions.

Findings of this study indicated that the IRT group profiled considerably higher than the TST group in the field of static balance, dynamic balance, and controlled stop-jump shooting task (all *p*’s < 0.001, |*d*| value between 0.778 and 1.090). The electromyographic results from the core muscles also indicated the internal dynamics underlying this complete adaptation. The recruitment intensities of the three primary core muscles (TrA, ES, and MF) in the IRT group became significantly improved (all *p*’s < 0.001, |*d*| value between 0.446 and 0.543). This observation supports the idea that instability resistance exercise not only stimulates the stimulation of a single core muscle but also supports the emergence of a new neuromuscular pattern of exercise control that employs multimuscle synergistic activation. This synergy is the core manifestation of “environmental constraints driving coordinated reorganization” in dynamic systems theory. For example, in a stop-jump shooting task, the TrA is activated in advance to maintain trunk stability; the ES contracts synchronously to resist forward leaning; and the MF finely regulates spinal segmental stability. Combining these coordinated motions in a more efficient kinetic chain enables athletes to respond quickly to changes in posture by coordinating the recruitment of core muscles in most dynamic actions, such as making a sharp turn at fast directional changes or making a sudden stop. As a result, both balance control and task-specific shooting improve at the same time.

This pattern of multimuscle synergy and multiperformance improvement implies that instability resistance training stimulates multivariable coordination optimization, a feature of non-linear learning. It allows an athlete to develop more refined balance measures and intermuscular coordination via repeated regulation and self-exploration within complex, dynamic settings to eventually maximize overall athletic performance (5, 29).

### 4.4 Integrated analysis: systematic training effects validated by multiple theoretical perspectives

From the integrated perspective of the three major theories, combined with the electromyographic results of the core muscles, the mechanism of instability resistance training can be summarized as follows.

Unstable environmental stimulation → enhanced sensorimotor integration → coordinated increase in core muscle activation → strengthened core stability and neuromuscular fatigue resistance → self-organization of movement coordination → optimization of balance and task-specific performance under the present testing protocol.

Improved core muscle recruitmentappears to be an essential intermediate within this model of systematic adaptation. It incorporates the neuro-regulatory actions of augmented sensorimotor integration, which translate multisensory feedback into accurate muscle contraction, and also provides the physiological basis for enhanced core stability through coordinated muscle action and enhanced control of posture. The process eventually allows the optimization of self-organized movement, which constitutes an entire regulatory circle across neural, muscular, and behavioral systems. The results of this paper are consistent with those reported in other studies (30, 31), further proving that balance ability plays a leading role as a basic motor capacity. This underscores the fact that instability resistance training has great potential for application in team invasion sports like basketball.

Also, the postexperimental 8-week test revealed a significant difference in absolute strength increase between the IRT and TST groups. Compared with the IRT group, members of the TST group demonstrated greater improvements in absolute strength, as indicated by the training logs. This can be attributed to the fact that mechanical tension in muscle fibers is the main determinant of maximal strength (32, 33). In instability resistance training, participants normally apply less force to the outside to maintain posture and prevent injury, leading to less overall mechanical tension compared to training on a steady surface; therefore, short-term improvements in absolute strength are restrained (3). Traditional strength training is typically more effective for maximizing external load, recruiting high-threshold motor units, and promoting muscle hypertrophy (33, 34), whereas instability resistance training places greater demands on intermuscular coordination, proprioceptive feedback, and postural control (3, 9, 35). These adaptations are thought to reflect neuromuscular and sensorimotor refinements rather than purely morphological changes (8, 32). As these two training approaches designate different adaptations, short-term training on a stable surface is more productive for increasing absolute strength. The present findings may be explained by the specific loading characteristics of instability resistance training and the physiological requirements for muscle hypertrophy. These results are consistent with Stanković et al. (36), who suggested that instability resistance training loads should not exceed approximately 50% of body weight; at such relatively low loads, training primarily promotes neuromuscular coordination rather than providing a sufficient stimulus for muscle hypertrophy (36).

Moreover, a slight decline was observed in the TST group's controlled stop-jump shooting task score after training, despite possible improvements in absolute strength. This finding suggests that increases in maximal strength may not directly transfer to shooting performance. In the short term, strength-oriented training may temporarily affect fine force control and movement coordination, thereby reducing force steadiness during skill execution (37). When the motor system is required to generate higher levels of force output, some precision in the system might be compromised, leading to reduced shooting accuracy. In addition, strength training can adjust the timing of upper-limb joint coordination and change the kinetic properties involved in the shot, which can further influence accuracy (38). Even though conventional strength training can boost explosive power, research findings show that increases in strength do not necessarily improve lower-limb motor control. Indicatively, high lower-limb muscular power in adolescent basketball players remains strongly correlated with knee valgus angles (39). Equally, in a study solely using traditional resistance-based training, no improvement in knee valgus was observed during vertical drop exercises (40). Conversely, balance control training can greatly reduce knee valgus and improve balance (41). Thus, the conventional approach to strength training does not prioritize improving core muscle recruitment synergy compared with its alternative, which results in compensatory responses to sudden stops (e.g., knee valgus and forward lean of the trunk) that distort the shooting technique and nullify the advantage of greater strength. Comparatively, the participants in the IRT group, with better core muscle recruitment, increased dynamic stability but retained fine motor control without the problem of increased strength at the expense of performance, which further supports the appropriateness of instability resistance training for improving task-specific execution under the present assessment conditions.

## 5 Conclusions

Theoretically, the research provides empirical support for three major theories of motor control. Under the sensorimotor control theory, increased TrA recruitment validates the mechanism of “sensory integration → core activation → static balance.” Within the core stability theory, enhanced ES and MF recruitment explains the relationship of “core control →  balance → task-specific.” According to dynamic systems theory, the coordinated recruitment of the three core muscle groups reflects the process of “environmental constraints → movement self-organization → fatigue-related late-set shooting accuracy.” Combined, these results demonstrate that instability resistance training can produce synergistic effects across multiple levels of neuromuscular control.

In practice, instability resistance training sessions be systematically integrated into college basketball programs and form part of conventional strength training, establishing an alternating process of training stability and instability. TST is also devoted to developing excellent strength, whereas instability resistance training also involves strengthening core muscle recruitment and dynamic stability. A complementary integration of the two approaches can more accurately enhance the overall performance capabilities of the athletes.

### 5.1 Limitations of the study

The intervention time of the study was relatively short, and the sample size was small, with low representativeness, as only 60 individuals participated. The study did not classify athletes by playing position nor explore possible differences in training effects across positions. The further timing analysis of core muscle activation and the frequency characteristics of the electromyographic signal were not included among the measurement indicators; thus, the dynamic characteristics of core control could not be further explored. All tests were carried out in non-competitive environments and were not simulated in competitive tests with high-intensity, multitask interference and other realistic game situations; thus, the applicability of the above conclusions in real competitive situations needs to be further verified. In addition, the stop-jump shooting task used in this study does not capture all determinants of in-game shooting performance. Therefore, the observed improvement was interpreted as task-specific rather than an overall improvement in basketball shooting performance.

### 5.2 Future research directions

Future research should consider adding time to the intervention and the cases, and cluster athletes by position (guard, forward, center), so that the information about differences between training positions can be examined using instability resistance training; expand the measurement metric system by incorporating core muscle activation timing, electromyographic frequency characteristics, proprioception tests, and neural response speed assessments to delve deeper into the physiological mechanisms underlying enhanced core muscle recruitment capacity; integrate real-game scenarios and multitasking conditions into testing protocols to validate the practical application value of instability resistance training in competitive settings; and explore optimal combination models between instability resistance training and traditional training to provide more actionable integrated solutions for basketball training.

## Data Availability

The original contributions presented in the study are included in the article/Supplementary Material, further inquiries can be directed to the corresponding author.
